# Geriatric surgery – present and perspective

**Published:** 2008-08-15

**Authors:** Popa Florian, Mihai Păduraru

**Affiliations:** * ”Carol Davila” University of Medicine and Pharmacy, Bucharest, Romania

**Figure:**
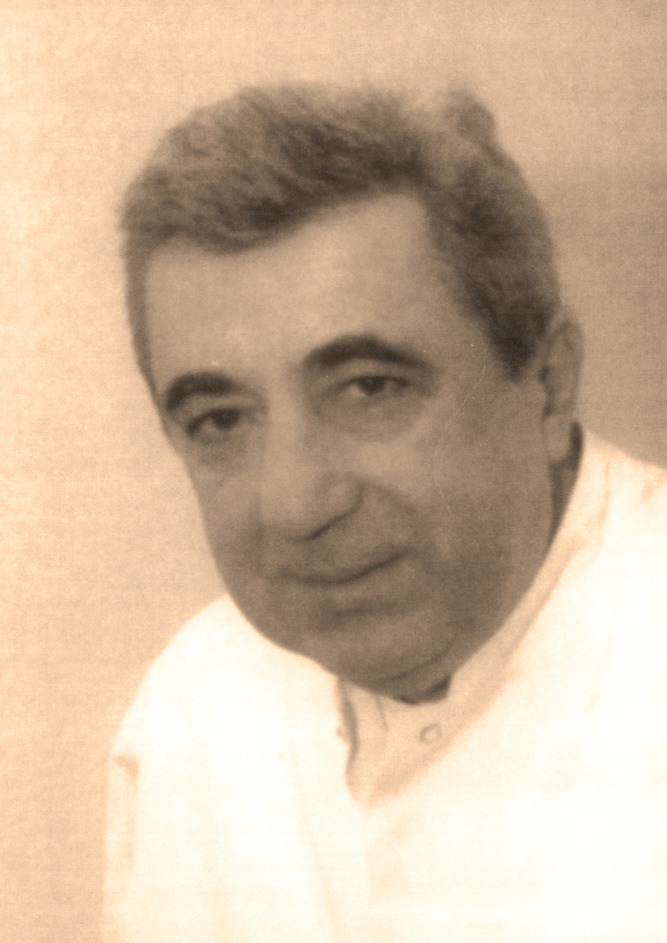


The trend at present planetary human society is restructuring throughout three fundamental and simultaneous processes with serious socio-economic implications, represented by globalization, urbanization and aging population.

This latter process reshuffled, the aging population, an inevitable progressive, at global scale phenomenon, new in the history of humankind, has, at the substrate, on one part the reduction in long-term birth, a process superimposed to the increase of longevity, and, on the other hand, the effectiveness of preventive and curative medical measures, important progresses made in the medical sciences domain, sustained by economic and industrial growth, as well as by the socio-cultural developments. All these led, in the second half of the XXth century, to an important extension of the life expectancy at birth.

The percent of elderly people reports a numerical growth, in terms of absolute, as well as relative scale, reported to the full extent of human resources. The ageing population, a process registered in all developed countries, and not only, is due to the reduction tendency of birth and fertility and in the same time, to the decreasing of universal morality. 

**The ageing process at global scale.** If the world's population was about one billion inhabitants in the year 1800, increasing barely to two billion by the year 1930, and having a progressive increase, reaching three billions in 1960, four in 1974, five in 1987 and six in 1999, the current figure exceeds 6.5 billion in 2005.

**Graphic No. 1:**
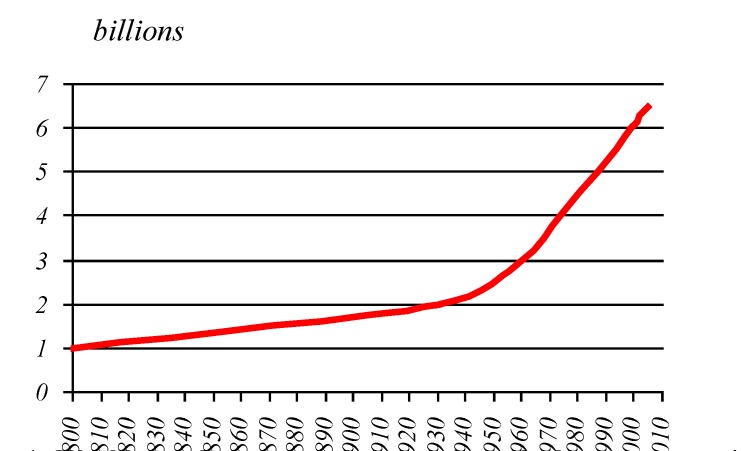
World population growth curve along the last two centuries

After this exponential growth of the population in the recent decades, forecasts for the XXIth century evaluates that the global population will continue to grow but in a slower pace exceeding the threshold of 9 billion in 2050. 

The ageing population presents a deeper growth; both in absolute figures, as well as reporting the active working population, estimating that, for the first time in the history of humankind number of elderly will be higher than that of children (age group of 0-15 years).

The number of people over the age of 60 years increased from 200 million worldwide during the year 1953, which represented 8% of the global population to over 600 million in recent years (10% of the population of 6 billion in the year 2000), forecast being of 21% in 2050 when ratings show a number of 2 billion elderly on the globe. 

**Graphic No. 2:**
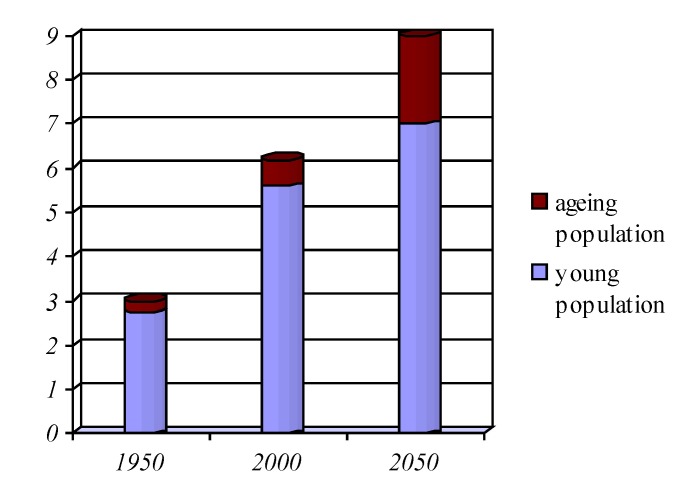
The share of older population in the population overall

The structure by age of the population in different countries is presented very divers, economically advanced countries having a ratio of 17% of older people, compared with only 7% in less developed regions of the world.

**Graphic No. 3:**
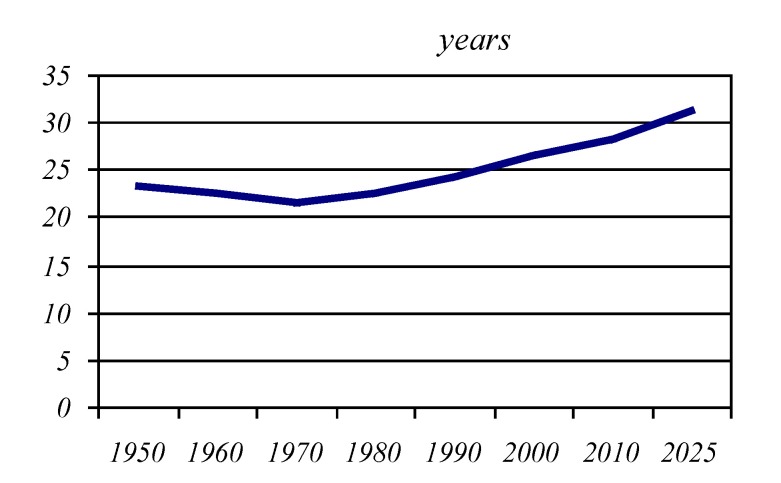
The median age of the population worldwide

**Graphic No. 4:**
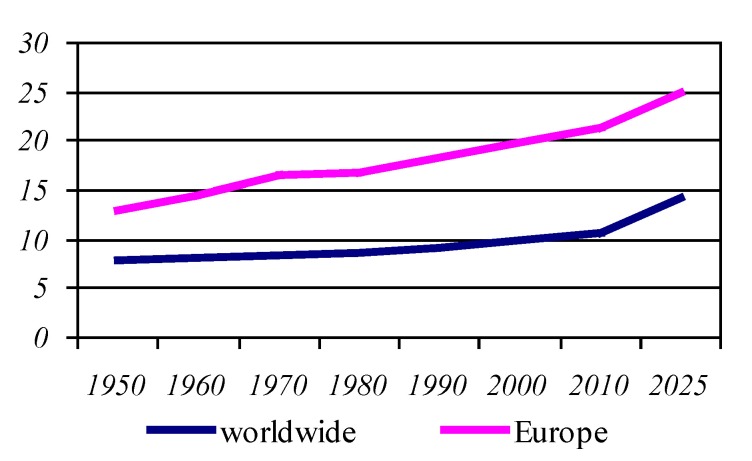
The proportion of elderly in Europe and the world

In conclusion, in a broad perspective, the global demographic dominant trend will be ageing population; this issue is clearly obvious by the increasing median age of the world's population from 21.6 years in 1970, to 26.5 years in 2000, being evaluated at 31.2 years in the year 2025. 

**Ageing population in Europe.**

"The most elderly", continent presents an average age of the population of about 1.3-1.4 times higher than that of the world population, being estimated for the year 2025 to approximately 40 years. 

The Europe population aged continuously after 1950, between 1950 and 1970 the proportion of elderly population raising from 13% to 17%, in the year 1985 the proportion of elderly is 18%, the ratings for the next decade showing an increase to 25%.

The enlargement of the European Union in 2004, from 15 to 25 members, brought an extra 74.3 million population, standing at 453 million inhabitants and with the accession of Romania and Bulgaria in 2007, added another 29.5 million inhabitants, the UE has thus become the third political entity in the world with 482.5 million inhabitants, after China (1.3 billion) and India (1.1 billion). The addition of the population comes on the background of a demographic decline that face both the old and the new members of the Union. 

**Graphic No. 5:**
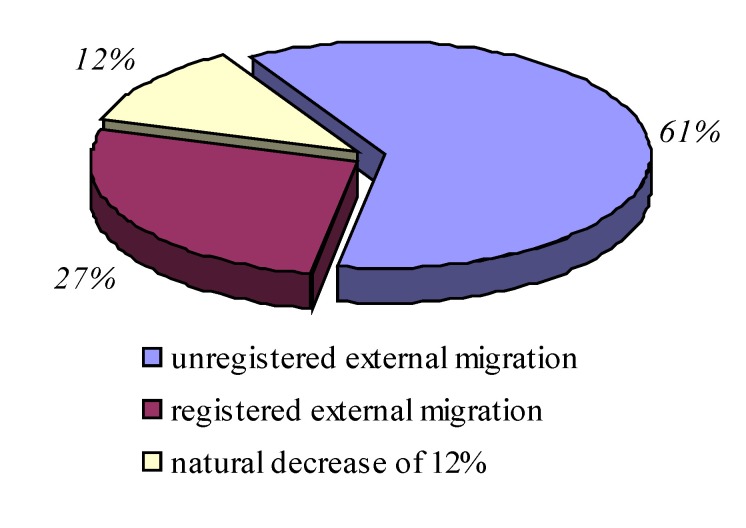
The components of population decrease

**Graphic No. 6:**
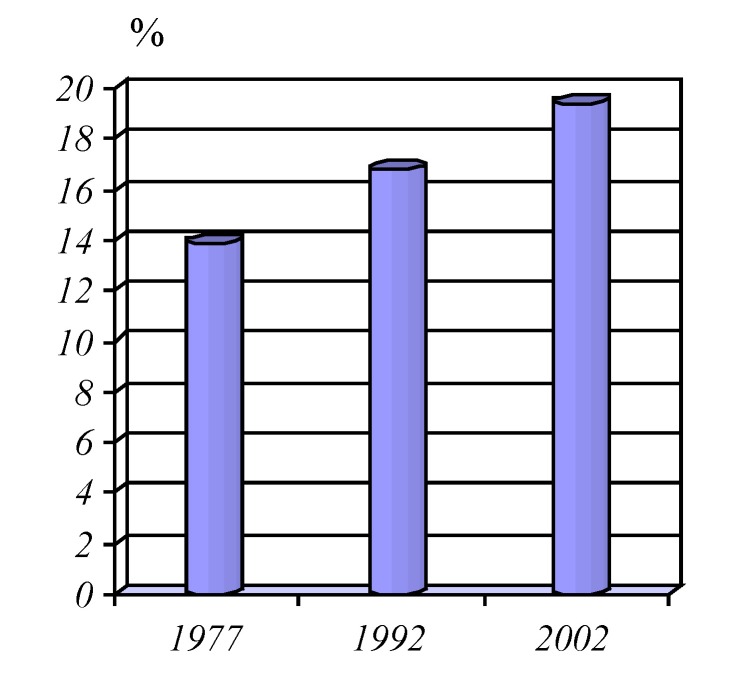
The share of population over 60

Generated in particular by the decrease of the level of fertility (in almost all member countries) and fast increasing life expectancy at advanced age (especially the EU-15, countries that focus almost three quarters of the current EU population) as a direct result of improving quality of life and medical progress (showed in a decrease in mortality rates), the process of ageing will increase in the future. 

**Demographic Ageing in Romania.** In the last decades in our country are highlights a demographic phenomenon with the main feature the ageing population, but unlike the evolution of the world population, we register a decrease of its number. With a complex causality, influenced by economic and social conditions, this phenomenon mostly concerns through its medium and long term consequences.

By comparing data last two censuses, one in 1992 when were registered 22,786,000 people, and in 2002 when were counted 21,680,974 people, we see a decrease in the country population with 1.1 million which represents approximately 5%. 

Components of the decrease in population were represented by external migration not registered statistic - about 700 thousand people representing 61%, natural decrease - 27% and external migration statistically recorded - 12% decrease in population by external migration addressing especially in young people. 

If at this decrease between the two censuses we add reduction of population that took place between 2002 and 2005, we reach a value of 1.5 million people, which represent about 7% of the country's population in 1990. 

In Romania share of the population aged over 60 years increased from 13.9% in 1977, to 16.8% in 1992 and 19.4% in 2002, the phenomenon is more accentuated in female population with a share of 21.7% compared with men - 16.9% , the ageing population is also better represented in rural areas where it is valued at nearly one quarter. 

It also noted that while worldwide the global population has a marked growth trend, in Romania population has declined over the past ten years, by a growing percentage and in absolute number of older people. 

All the above elements leading to the conclusion that Romania has an aging population with a tendency emphasis on the phenomenon. 

**Demographic prospects in Romania.** If currently, of the 21.6 million inhabitants, 10.5 million are adults, 5 million - young and children, and 6 million are elderly, over 50 years through an evolving with current parameters ,demographic picture will look completely different; elderly will represent more than half the population of the country, the number of adults and children will decrease, age pyramid-narrowing significantly it’s base. 

Therefore, the ageing process will intensify, consequences being - increase general fatalities rates, increased share in the elderly population, low sustainability of health insurance systems, of pension systems, demographic decline obtaining wider dimensions. 

Demographic cost will be much stronger felt across the years 2025-2030, when the generations born after 1989, reduced in number, will be active population of the country, and will have to support an economic and social disproportionately large elderly population with specific health problems.

**Medical-surgical implications of ageing populations.** Following a broad representation in the population of elderly - patients aged over 65 years according to the WHO classification, they occupy an important place between admissions in surgical wards. In our Clinic representing 22.1% of total admissions and 20.47% of the cases who suffered surgery, assigned by percentage according to age categories - **Graphic No. 7**.

**Graphic No. 7:**
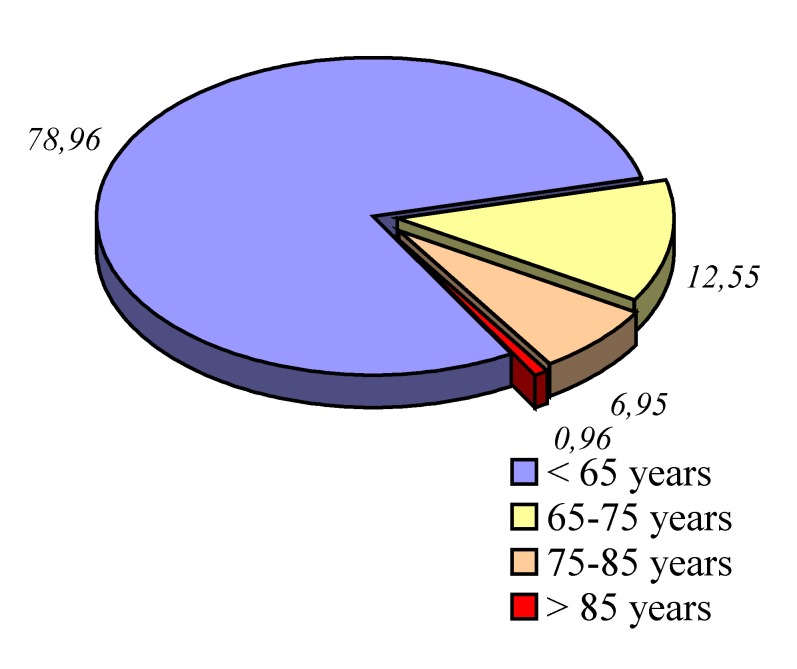
Graphic representation of the ratio of patients operate age

**Graphic No. 8:**
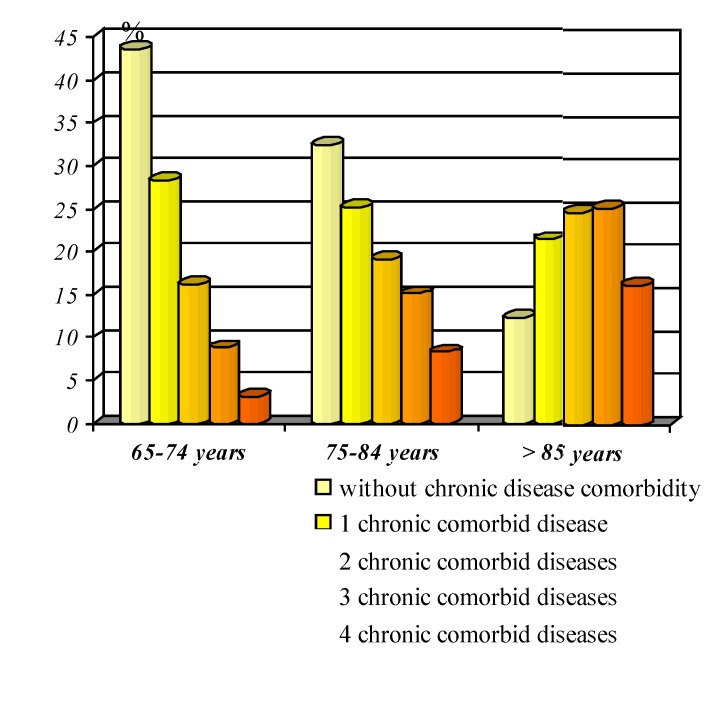
Representation percentage of cases with and without conditions comorbide

The clinic and laboratory evaluation of elderly patients reveal the existence of complex medical-surgical pathology in most cases, in a large part encountering even multiple morbidity associated. 

In a retrospective analysis on decades of age, I have noticed an increasing percentage of the elderly, along the three age groups, of cases where there were found comorbide affections - 56.4% in 65-74 years, 67 , 56% at 75-84 years and 87.55 over 85 years. Also, along with the submission elderly, is observed an increasing share of cases with 2, 3 and 4 impairments associated comorbide affections (**Graphic No. 8**), cardio-vascular diseases occupying the most important group of medical conditions associated with the elderly patients, consignment studied representing about 70% of 65-74 years, 73% of 75-84 years and 82% over 85 years. 

The elderly's comorbidities that are associated to the disease requireing surgery, appear often in the form of a pathological complex, which is manifested not only as an arithmetic sum of each disease, but with close pathologic interconnections with empowering effect between the pathological entities and with important implications for therapeutic opportunities and the anesthezic-surgical risk, implicitly affecting prognosis. 

This phenomenon highly increases the complexity of the cases, requires an interdisciplinary evaluation and application of vaste therapies that address all pathology components and their adaptation to the disease's stage and elderly patient's status. It also records an extension of recovery time and a dramatic alteration of prognosis, with the increased risk of complications occurrence (**Graphic No. 9**).

**Graphic No. 9:**
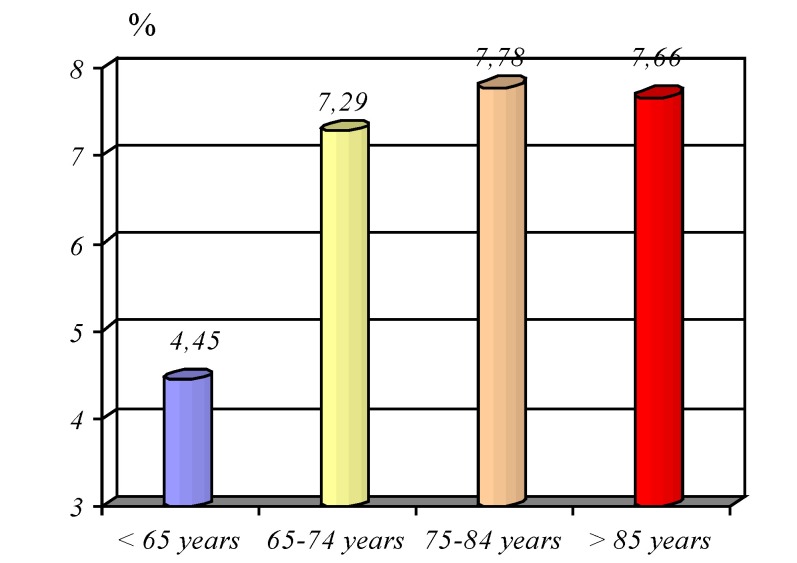
The average period of hospitalization age

**Graphic No. 10:**
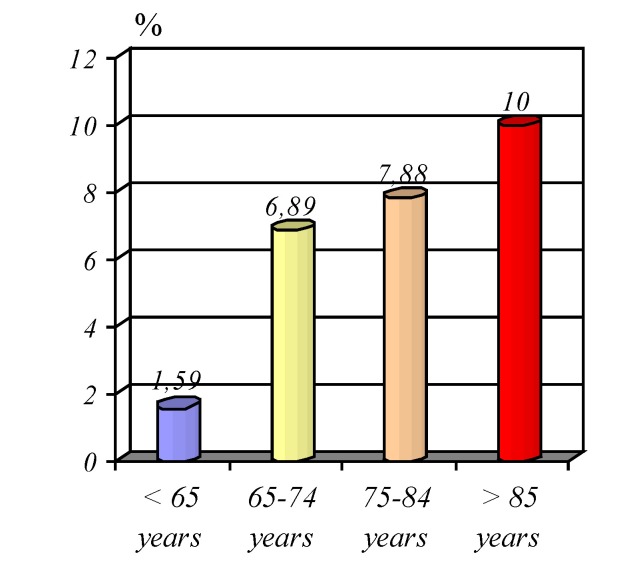
Share pathology digestive malignancies by age

Another observation is that related to the onco-geriatric pathology, the neoplastic disease of the digestive system in relation to benign pathology for admitted patients aged under 65 years and the elderly, recording a gradual and constant increase in the percentage of neoplastic diseases for the three categories age over 65 years (**Graphic No. 10**).

The rate between small - medium-scale and large-scale interventions for patients under 65 years is 45%/55%, while for the elderly patients is 17%/83% the balance leaning more towards large-scale interventions.

An analysis of the surgery applied to elderly patients and of the degree of emergency, has shown an increasing percentage of cases in which it has been intervened to forms of acute abdomen: 26.5% at 65-74 years, 34.52% from 75-84 years and 56.41% for patients over 85 years (**Graphic No. 11**).

**Graphic No. 11:**
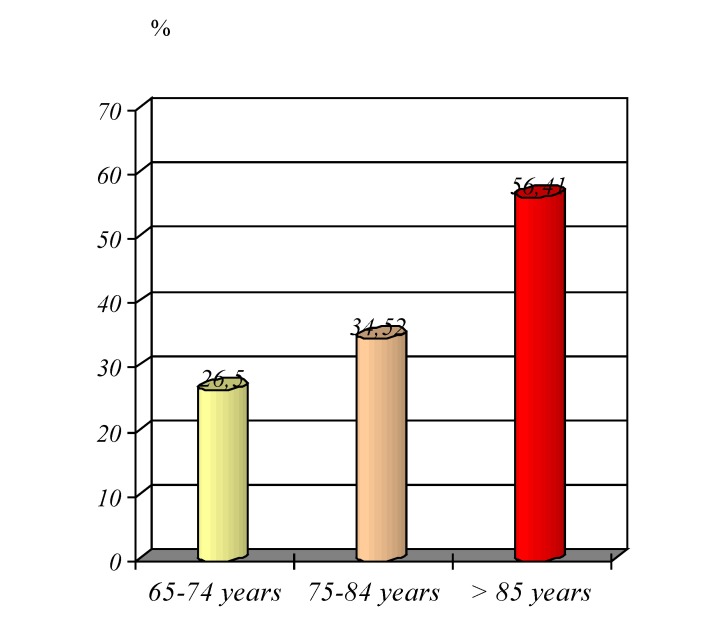
The share of cases of acute abdomen operate between cases

In comparison with the adult patients lot, the elderly are recorded and a numer of different forms of acute abdomen by etiology, this being different from one age to another in the lot of patients over 65 years old (**Graphic No. 12**).

**Graphic No. 12:**
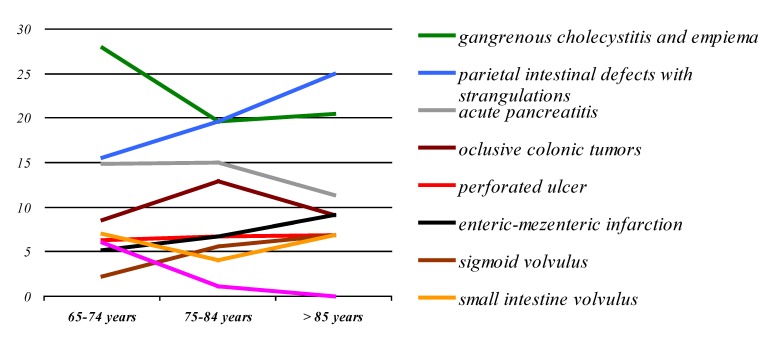
Graphic representation developments weights forms the acute abdomen in the elderly the three age groups: 65-74, 75-84 and over 85 years

he calculated death rate was very different and much higher for the older categories of age, so that patients between 65 and 74 years of age, it has a value of 4.96%, for those over 75 and under 85 years, a value of 10.07%, for pacients over 85 years the mortality was recorded to be 25.71%, which increased the interest in structuring by age and revealing at the same time a post-therapeutic evolution fundamentally different in relation to age (**Graphic No. 13**).

**Graphic No. 13:**
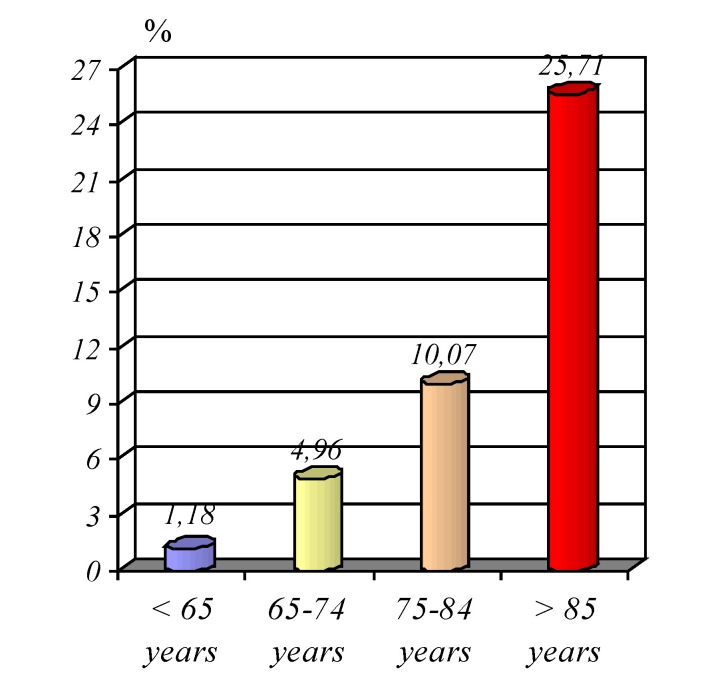
Graphic representation of mortality by age

In conclusion, and as a result of extensive representation of the elderly in the population, and with and ascending trend, patients over the age of 65 years represent a significant category among admissions in surgical wards.

The clinic and paraclinic assessing of elderly patients reveals the existence, in the vast majority of cases, of pathological complex, medical or surgical, and a wide area of pacients present an associated polimorbidity.

The division on three elderly patients age (65-74 years, 75-84 years and over 85 years), reveals important differences between the categories of age, both in terms of pathology, previosion and post-therapeutic developement, therapeutic attitude for the elderly sick imposes as judicious and variated, being correlated both with the status of the elderly (seriousness of the condition with surgical visa number and degree comorbide diseases) as well as the age category of the pacient.

Interdisciplinarity in geronto-surgical cases is a rule, cardiology consults and intern consults are musts.

Results of post-treatment and the hospital are so different from those of patients under the age of 65 years, and in the lot from an elderly age category to another.

**The prospects of geriatric surgery in our vision**

In the light of the above outlined and exposed by the particularities of elderly patients seen from the surgical perspective, but also for an optimization of therapy towards best decisions and best results, consider appropriate a special concern for geronto-surgery.

This could be materialized through the achievement of certain departments in existing clinics for surgery or by the creation of special sections, and in terms of improvement of personnel, through the establishment of over-specialization in the area, so that doctors become familiar with the whole issue of elderly surgical patients.

Through an analisys of the post-treatment evolution at elderly pacients, we consider the most probable segment for treatment by this over-specialisation is represented by pacients over 75 years, including thus the last two age categories according to the W.H.O., while the segment of 65-74 years would be one of interference with the adult surgery. Having the precedent of setting up the first National Institute of Geriatrics and Gerontology in the world (1952) by Acad. Prof. Dr. Ana Aslan, with the aim of research and treatment of specific medical problems for the elderly, i consider such a project achievable.

In support of this proposal wecall three argument categories, identified by us as medical-surgical, demographic and administrative organization. 

*Medical-surgical arguments:*

- Surgical pathology profile of the elderly with all its features - the increased share of cancer surgery (surgery onco-geriattrics), the specific forms that elderly patients present, the seriousness of injuries etc.

- most patients presenting their comorbide affections in different degrees of compensation - associated polimorbidity.

- Specific developments of elderly patients with higher rates of mortality in relation to younger patients that get exacerbated as patients fall into higher age groups.

- Higher rate of complications (morbidity) on one hand of the diseases (decompensations) and on the other of the surgical operations.

Demographic arguments

- The share of the current elderly population is increasing and the prospects of demographic analysis indicates a phenomenon of even more ageing of the population at least for the next decade.

- comparing to the establishment of paediatric Chirurgiei at the beginning of the XX century when the population structure was a pyramid-type (with many children and few elderly) and analyzing the current population structure in which the the percentage of elderly is growing and even exceeding the number of children, we distinguish the premises for creating such specialties in training / over-specialization: geriatrics Surgery. 

Also through a comparative analysis of the Nomenclature of medical specialties and the surgery reveals an asymmetry among medical specialties like geriatrics and gerontology. 

Administrative arguments

- The average length of hospitalization, increased to elderly patients and greatly extended to operated elderly patients. The health insurance system requiring a series of performance criteria with stipulated minimum, average and maximum hospitalisation periods, they may not be optimally kept in a clinic that deals with and which operates sick elderly.

- The interdisciplinary principle that must be applied to all elderly patients requires a number, an availability and a variety of specialists who could be much better trained in a specialized service.

- Through slower post-treatment evolution, through environment dependency and increased care need in wards with elderly patients, and the longer operate with the elderly,there is a necessity for more qualified personnel.

To these arguments we may add an aspect of elder's psychology, that the elderly will be able to socialize more easily in a department dedicated to their treatment.

Similar concerns and programs of excellence in the direction of geriatric surgery have been noticed in other prestigious universities and surgical societies, stressing the intrinsic value of the theme: North Shore University Hospital - Long Osland Jewish Medical Center - Surgery Program of Excellense - Geriatric Surgery (http://www.northshorelij.com/body.cfm?ID=3931), The Univerity of Oklahoma - Program of Geriatric Surgery http://w3.ouhsc.edu/surgery/Geriatric_Surgery.asp), Societa Italian di surgery - Italian Società di Chirurgia Geriatrica (http://www.sichirurgia.org/).
